# Supplementary figures and images for: Combined Metabolomics and Transcriptomics Analysis of the Distribution of Flavonoids in the Fibrous Root and Taproot of Polygonatum kingianum Coll.et Hemsl
Source: Genes (Basel). 2024 Jun 22;15(7):828. doi: 10.3390/genes15070828 (PMC11275391; doi:10.3390/genes15070828)

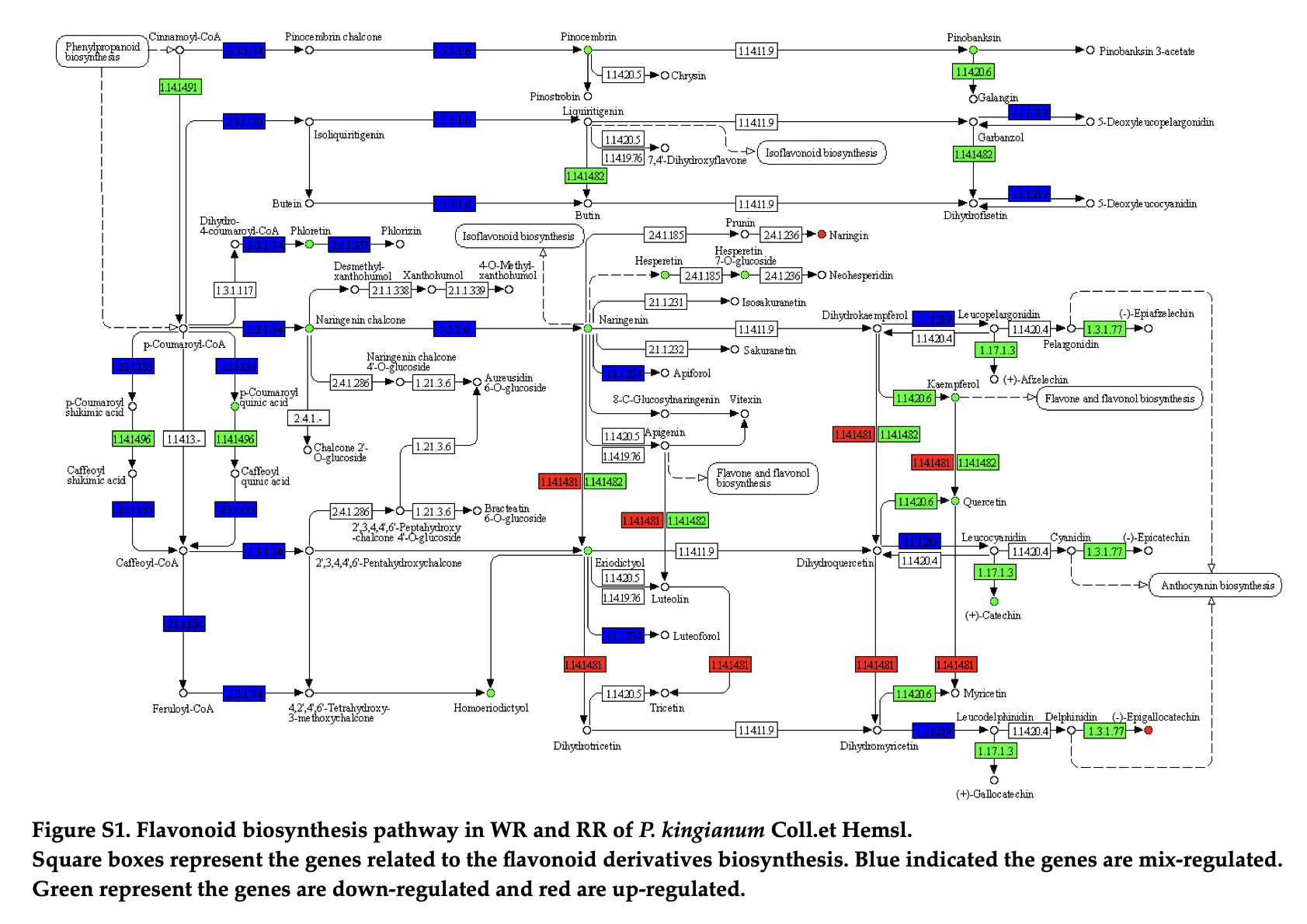

Supplement: Supplementary file 1 [file genes-15-00828-s001.zip › Figure S1.png]
